# Clinical Applications and Benefits of Using Closed-incision Negative Pressure Therapy with Novel Dressing: A Review Article

**DOI:** 10.7759/cureus.6902

**Published:** 2020-02-06

**Authors:** Dennis Adjepong, Bilal Haider Malik

**Affiliations:** 1 Neurological Surgery, California Institute of Behavioral Neurosciences and Psychology, Fairfield, USA; 2 Internal Medicine, California Institute of Behavioral Neurosciences and Psychology, Fairfield, USA

**Keywords:** surgical site infections, novel dressing, closed incision, postoperative wounds, negative pressure wound therapy

## Abstract

Surgical site occurrences (SSOs) are common in patients undergoing operative procedures, especially in the form of surgical site infections (SSIs). Multiple studies show that obesity, tobacco use, prolonged surgical time, and diabetes mellitus are the major risk factors for SSIs. SSIs increase healthcare costs and often result in morbidity. Many surgeons are currently using closed-incision negative pressure therapy (ciNPT) to counter SSIs. This method makes it easier for them to manage closed and surgical incisions. This technique has already been applied in the plastic surgery field. This study discusses how the use of ciNPT is helping surgeons to reduce complications related to SSOs. The technique has been reported to minimize the rate of reoperations, readmissions, and other wound-related complications. Using ciNPT with novel dressing has proved to be a significantly effective clinical intervention method in managing clean and closed wounds. The novel dressing protects the incision from external contamination and minimizes lateral tension.

## Introduction and background

Managing chronic and complex wound etiologies can be difficult and cause tremendous economic burden to healthcare organizations [1]. The situation presents the need to introduce innovative wound management strategies [2]. Complex wounds negatively affect patient mortality and morbidity, thereby causing lengthy hospital stays [3]. Due to the difficulty in treating and managing wounds, it becomes necessary for surgeons to be well-versed with the management of such patients and the comorbid conditions [4]. The use of a new dressing method in wound care is reported to be effective in enhancing the reparative process [5]. Closed-incision negative pressure wound therapy (ciNPWT) with novel dressings is divided into three phases, i.e., inflammation, remodeling, and proliferation and tissue formation [6]. This novel closed-incision management (CIM) has become a convenient method for negative pressure wound therapy (NPWT), with the clinical advantage of helping patients with postoperative complications [7]. This study investigates the clinical benefits and advantages of using ciNPT for wound dressing by examining the findings and results of the existing studies.

A traditional review of the existing studies was used as the primary method for data collection. Information was obtained from the medical database using keywords such as novel dressing, postoperative wounds, and negative wound therapy.

## Review

Intrinsic and extrinsic wound healing processes

Both natural and extrinsic factors impair the novel dressing in the wound. External factors include trauma or mechanical stress applied to the injury. Wound healing is strictly regulated by cytokines and growth factors released at the wound site [8]. Regeneration is uncommon, although there are hopes of forming a similar structure with the parent tissue [9]. Healing results in functionality and structurally satisfactory but not identical results [10]. Alterations in the wound by the growth factors and cytokines might disrupt the healing process and extend danger to other tissues. Wound infection therapy can be performed by using topical antibacterial medications, antiseptics, and system antibiotics [11]. There is no current information about the genetics of the disease. The literature is devoid of any data regarding the correlation between gene resistance and increased risk of surgical site infections (SSIs).

Each phase in the novel dressing is characterized by complex interactions between blood-borne types, cell types, extracellular matrix, and growth factors [12]. The phases do not represent distinct and separate events but are recognized as continuous and overlapping. Novel methods such as silver-containing dressing and photodynamic therapy enable the eradication of bio-film and multidrug-resistant bacteria [13]. Optimized methods for the prevention and detection of biofilm formation may lead to successive chronic wound care. Recent studies show that a better understanding of the cellular and molecular components in the patient’s body improves the diagnosis and tailored therapy [14].

Recent research supports the application of ciNPT among patients with SSI risk factors, including those with prolonged surgical time, tobacco use, diabetes mellitus, and obesity [15]. The use of ciNPT has been shown to be effective by benchtop and computer finite element studies, which explain how closed incisions protect the wounds from external contamination [3]. The introduction of ciNPT in managing wounds has introduced a new scientific and clinical approach that utilizes innovative technologies to revolutionize wound care [5].

Currently, surgeons are using ciNPT in various clinical activities. ciNPT can manage surgical incisions by reducing edema, line tension, and by the provision of an airtight seal [13]. The method is beneficial in the prevention of wound incision complications. The effects of ciNPT are divided into intermediate, immediate, and long-term ones. It protects incision from SSIs and external contamination [15]. The intermediate effects include reduced edema, increased lymphatic flow, and seroma formation [16]. Surgeons have always been interested in treating wounds using negative pressure wounding techniques, which eventually led to the emergence of ciNPT [17]. Patients always consider cost when it comes to choosing a healthcare facility [18]. ciNPT is a cost-effective treatment in managing gynecological cancers as it reduces rates of SSI [19]. The table below (Table 1) clearly shows that ciNPT minimizes the frequency of reoperations, readmission, and other wound-related complications.

**Table 1:** Studies that discuss the clinical benefits of closed-incision negative pressure therapy ciNPT: closed-incision negative pressure therapy; SSIs: surgical site infections

No of studies	Author name	Year of publication	Country of origin of the study	Inference
1	Wilkes et al. [20]	2012	USA	Using ciNPT enhances the normalization and reduction of bolster apposition forces and tissue stresses
1	Fernandez et al. [5]	2019	USA	Using ciNPT immediately during the postoperative period prevents risk complications and reduces the cost of care
1	Reddy [21]	2016	USA	Using ciNPT had favorable results over other methods in reducing the risk of complications among post-sternotomy patients
1	Kwon et al. [22]	2018	USA	Using ciNPT resulted in reduced readmission and reoperation risk among a group of patients with high-risk of SSIs

Economic cost: ciNPT versus without ciNPT

The use of ciNPT has helped to significantly reduce healthcare costs in many cases. This is associated with the accuracy of the process, including lowering site complications on the patient's surgical site during the postoperative period. Studies have also shown how surgical disciplines have enhanced safety among patients with integrated effectiveness of ciNPT. In the United States, the cost of savings has increased in the past decade. The table below (Table 2) clearly points to the massive healthcare savings as a result of adopting ciNPT.

**Table 2:** Studies that discuss the economic benefits of closed-incision negative pressure therapy

Year	Author	Healthcare cost savings	Inference
2014	Murphy et al. [23]	$5,400 per person	Saves up to $64,000 annually
2015	Bonnar et al. [2]	$7,200 per person	Saves up to $86,400 annually
2016	Chopra et al. [4]	$2,745 per person	Saves up to $32,940 annually
2017	Fernandez et al. [7]	$5,705 per person	Saves up to $68,460 annually
2018	Kwon et al. [22]	$6,045 per person	Saves up to $72,540 annually
2019	Gabriel et al. [1]	$1,616 per person	Saves up to $19,392 annually

Again, with improved technology, the estimated costs of ciNPT are expected to decrease over the next decade [24]. More advancements are expected to emerge, given the anticipated enhanced technology coming up in the next decade.

Clinical applications and analysis of ciNPT

Experiments and experiences with CINPT have proved useful in the development of a consensus among panels constituted by surgeons [25]. It is estimated that over the past decade, not using ciNPT has increased the cost of treatment for each patient, given the more extended hospitalization stays. On average, each patient had to shell out a minimum of $50,000 in fees, with the hospitalization stays lasting between 9.7 and 13.7 days [16]. Cost analysis has shown that the overall economic saving using ciNPWT is approximately $11,300 for every patient [17]. Around the world, ciNPWT improves the quality of life as it increases accuracy during the diagnosis and treatment of patients, both in the short and long run. Advancement in healthcare generally is expected to increase the efficiency of NPWT, ensuring that novel dressing improves the quality of life for all patients in the next decade.

Environmental factors affecting ciNPT

Many environmental factors have affected ciNPWT negatively [26]. These include extreme cold and warm weather [22]. Healthcare practitioners are required to ensure that they are aware of the environmental/climate-related peculiarities of the region where they practice ciNPT. These include extreme weather conditions and earthquakes. In the case of earthquakes, practitioners are required to ensure that all patients are in a safe place. In extreme cold and heat weather, patients' therapy rooms must remain in the required temperature for the successful access to and healing of the wound.

Benefits of ciNPT with Novel Dressing

There are many benefits of NPWT compared to other traditional forms of therapy. NPWT is considered advantageous given its faster effects in skin healing, including on transplanted skins. Overall, NPWT has drastically reduced lengthy hospital stays and ensured a quicker growth of tissues guaranteeing the skin's thickness [15]. Again, all stakeholders benefit from the increased use of NPWT, given its effectiveness in reducing healthcare costs and improving the quality of care at healthcare centers. Surgeons, patients, insurance companies, and nursing staff all benefit from NPWT (patent citation: inventors: Wu K, Hu D, Nag S; assignee: Spiracur Inc. Methods and devices for applying closed incision negative pressure wound therapy. The United States patent: US 8,444,614; https://patents.justia.com/assignee/spiracur-inc). Surgeons and healthcare staff benefit from faster patient recovery, with patients and insurance firms incurring lower costs during hospitalization [27]. 

Unanswered questions

There is still a need for further studies to address questions regarding the effectiveness of using ciNPT and its safety when applied in various patient populations [28]. There is also a need for further studies that involve a more detailed comparison between the efficacy of ciNPT and standard therapies on patient outcomes and wound healing.

## Conclusions

This study proves that the use of ciNPT is effective in reducing complications related to SSIs. The method minimizes the rate of reoperations, readmission, and other wound-related complications. Using ciNPT with novel dressing has proved to be a significant clinical and operative intervention method in managing clean and closed wounds. SSIs increase healthcare costs and often results in morbidity. The novel dressing protects the incision from external contamination and minimizes lateral tension. It also helps to normalize stress distribution, decrease lateral pressure on the incision, and increase appositional strength. We recommend that surgeons consider using ciNPT more frequently on patients undergoing procedures with a potential for high morbidity rate due to SSIs.
